# Sunlight-induced rapid and efficient biogenic synthesis of silver nanoparticles using aqueous leaf extract of *Ocimum sanctum* Linn. with enhanced antibacterial activity

**DOI:** 10.1186/s13588-014-0018-6

**Published:** 2014-12-29

**Authors:** Goutam Brahmachari, Sajal Sarkar, Ranjan Ghosh, Soma Barman, Narayan C Mandal, Shyamal K Jash, Bubun Banerjee, Rajiv Roy

**Affiliations:** 1 Department of Chemistry, Laboratory of Natural Products and Organic Synthesis, Visva-Bharati (a Central University), Santiniketan, 731 235 West Bengal India; 2 Department of Botany, Microbiology and Plant Pathology Laboratory, Visva-Bharati (a Central University), Santiniketan, 731 235 West Bengal India

**Keywords:** Silver nanoparticles, Ocimum sanctum, Sunlight, Antibacterial activity, Mode of action, Nanomedicine

## Abstract

**Background:**

Nanotechnology is now regarded as a distinct field of research in modern science and technology with multifaceted areas including biomedical applications. Among the various approaches currently available for the generation of metallic nanoparticles, biogenic synthesis is of increasing demand for the purpose of *green nanotechnology*. Among various natural sources, plant materials are the most readily available template-directing matrix offering cost-effectiveness, eco-friendliness, and easy handling. Moreover, the inherent pharmacological potentials of these medicinal plant extracts offer added biomedical implementations of the synthesized metal nanoparticles.

**Results:**

A robust practical method for eco-friendly synthesis of silver nanoparticles using aqueous leaf extract of *Ocimum sanctum* (Tulsi) as both reducing and capping agent, under the influence of direct sunlight has been developed without applying any other chemical additives. The nanoparticles were characterized with the help of UV-visible spectrophotometer and transmission electron microscopy (TEM). The prepared silver nanoparticles exhibited considerable antibacterial activity. The effects were more pronounced on non-endospore-forming Gram-positive bacteria viz., *Staphylococcus aureus*, *Staphylococcus epidermidis*, *and Listeria monocytogenes* than endospore-forming species *Bacillus subtilis.* The nanoparticles also showed prominent activity on Gram-negative human pathogenic *Salmonella typhimurium*, *Escherichia coli*, *Pseudomonas aeruginosa*, and plant pathogenic *Pantoea ananatis.* A bactericidal mode of action was observed for both Gram-positive and Gram-negative bacteria by the nanoparticles.

**Conclusions:**

We have developed a very simple, efficient, and practical method for the synthesis of silver nanoparticles using aqueous leaf extract of *O. sanctum* under the influence of direct sunlight. The biosynthesis of silver nanoparticles making use of such a traditionally important medicinal plant without applying any other chemical additives, thus offers a cost-effective and environmentally benign route for their large-scale commercial production. The nanoparticles dispersed in the mother solution showed promising antibacterial efficacy.

**Graphical Abstract:**
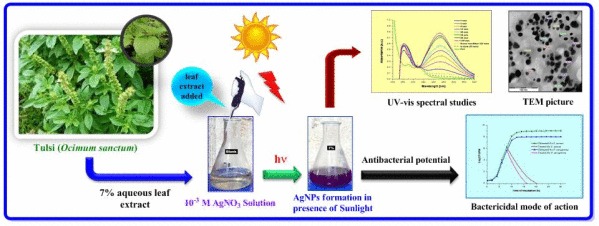
Sunlight-induced rapid and efficient biogenic synthesis of silver nanoparticles using aqueous leaf extract of *Ocimum sanctum* Linn. with enhanced antibacterial activity.

**Electronic supplementary material:**

The online version of this article (doi:10.1186/s13588-014-0018-6) contains supplementary material, which is available to authorized users.

## Background

Nowadays, nanotechnology is regarded as a distinct field of research in modern science and technology with multidirectional applications [1]-[7]. Useful application of nanotechnology in medicinal purposes is currently one of the most fascinating areas of research. Metallic nanoparticles (NPs) have also been receiving considerable interest in biomedical applications [1],[8]; silver nanoparticles (AgNPs), in particular, are finding applications to the researchers as tools for antibacterial and antifungal [8], anti-inflammatory [9], wound healing [10], radio-imaging, retinal neovascularization [11],[12], antiviral and antioxidant [13] agents, and also as novel cancer therapeutics, capitalizing on their unique properties to enhance potential therapeutic efficacy [11],[12]. In an *in vivo* experiment, silver oxide nanoparticles were found to exhibit notable antitumor efficacies in transplanted Pliss lymphosarcoma tumor models when administered by intravenous injection in the form of aqueous dispersions [14]. Interest in the clinical uses of silver nanoparticles has been facilitated due to their rapid availability as well. The diameters of AgNPs are normally smaller than 100 nm and contain 20 to 15,000 silver atoms per particle [15],[16]. Thus, when cells or tissues get exposed to AgNPs, the active surface area of the nanoparticles become significantly large compared to that of the ordinary silver compounds. Such enhanced surface area of metallic nanoparticles is supposed to be responsible for exhibiting remarkably unusual physicochemical properties and biological activities [17]. It is also assumed that promising inhibitory function of AgNPs originates from their interactions with sulfur-containing proteins as well as with phosphorus-containing compounds like DNA inside the microbial membranes [1]. Besides, silver-embedded fabrics are now used in textile industry in manufacturing sporting equipments [18].

Among the various approaches currently available for the generation of metallic nanoparticles, biogenic synthesis that avoids the use of toxic and hazardous chemicals is of increasing demand for the purpose of *green nanotechnology*. It is well-established that biological efficacies of synthesized nanoparticles largely depend on the nature and concentration of capping agent(s) used for stabilizing the nanoparticles. Several matrixes for the biogenic synthesis of such nanoparticles are reported so far, and they include microorganisms such as bacteria [19], fungi [20], enzymes [19], and useful medicinal plant extracts [4],[17],[21],[22]. Among these natural sources, plant materials are the most readily available template-directing matrix offering cost-effectiveness, eco-friendliness, and easy handling much suitable for scaling up processes. Uses of plant materials in generating metallic nanoparticles in both micro- and macroscales are, thus, more advantageous over the microorganism-based methods involving complicated and sensitive cell culture processes. Moreover, the inherent pharmacological potentials of these medicinal plant extracts offer added biomedical implementations of the synthesized metal nanoparticles [23]-[25].

In compliance with this view, varying extracts of a huge number of medicinal plants such as *Azadirachta indica*[23], *Boswellia ovalifoliolata*[26], *Carica papaya*[27], *Catharanthus roseus*[28], *Cinnamomum camphora*[21], *Citrus aurantium*[29], *Datura metel*[30], *Jatropha curcas*, *Medicago sativa*[31], *Nelumbo nucifera* (lotus) [32], *Pelargonium graveolens*[33], *Solanum melongena*, *Tridax procumbens*, *etc*. have already been used to synthesize and stabilize metallic nanoparticles, very particularly silver (Ag) and gold (Au) nanoparticles [4],[19]. However, a little has been carried out on engineering approaches, viz. rapid nanoparticles synthesis using plant extracts and size control of the synthesized nanoparticles [23],[29]. Besides, the uses of edible plants are in tremendous demand for the biomedical applications of AgNPs.

In ancient Ayurveda, ‘Tulsi’ (*Ocimum sanctum* Linn., family: Limiaceae) is known as *the elixir of life* since it promotes longevity and is used in many formulations for the prevention and cure of various ailments [34]. All parts of the plant such as fresh leaves, juice, seeds, and volatile oil are very beneficial to us. The *O. sanctum* plant finds wide applications in the treatment of cough, coryza, hay asthma, bronchial infections, bowel complaints, worm infestations, and kidney stones in traditional systems of medicine [35],[36]. *O. sanctum* possesses diverse pharmacological properties that include antioxidant [34], antibiotic, antidiabetic, antiatherogenic, immunomodulatory [34],[37], anti-inflammatory [9],[37], analgesic, antiulcer [37], and chemo-preventive and antipyretic properties [38]. Tulsi leaf extract reduces blood glucose and cholesterol and promotes immune system function [39], and one of the constituents, β-elemene, has been reported to have potent anticancer property [40]. The major phytochemicals present in *O. sanctum* plant belong to terpenoid, phenolic, tannin, steroid, alkaloid, and saponin class of compounds [41].

That is why *O. sanctum* plant has recently drawn an attention for its possible uses in the biogenic synthesis and stabilization of metal nanoparticles [1],[8],[42],[43]; however, these reported methods suffer from certain shortcomings such as the use of other chemical additives and heating conditions. Hence, development of environmentally more benign, cost-effective, and efficient methodology for the rapid biogenic synthesis of nano-sized metal particles under mild reaction conditions using medicinally significant and edible *O. sanctum* plant as both metal ion reducing and good capping agent is still warranted. We, herein, wish to report for the first time a simple and efficient one-step protocol for the rapid synthesis of AgNPs (7 to 11 nm) by reducing Ag^+^ ions in aqueous silver nitrate solution using aqueous fresh leaf extract of *O. sanctum* in the absence of any chemical additive under direct sunlight irradiation with excellent stability. We compared the efficacy of *O. sanctum* with another two medicinal plants, *Citrus limon* Linn. (family: Rutaceae) and *Justicia adhatoda* Linn. (family: Acanthaceae) under the same conditions. Besides, the enhanced antimicrobial activity of the AgNPs plant leaf extract (PLE) against some known pathogenic strains is also evaluated.

## Methods

### General experimental procedures

Fresh leaves of three medicinal plants Tulsi (*O. sanctum* Linn.), *C. limon* L., and *J. adhatoda* L. were collected in October 2013 at and around Santiniketan, West Bengal, India, and identified by Dr. H. R. Chowdhury (Botany Department, Visva-Bharati University). Voucher specimens are preserved in the Laboratory of Natural Products and Organic Synthesis of this university. The water used as the solvent was previously subjected to deionization, followed by double distillation (first time in alkaline KMnO_4_). Fresh leaves of the three plants were washed thoroughly with double-distilled water for several times to make it free from dust and were then cut into small pieces. Silver nitrate (AgNO_3_) (Sigma-Aldrich, Bangalore, India) were used as the source of Ag(I) ion required for the synthesis of Ag nanoparticles. UV-vis absorption spectra were recorded on a Thermo Scientific Spectrascan UV 2700 1 nm double beam spectrophotometer (Thermo Fisher Scientific, Waltham, MA, USA). Sample for transmission electron microscopy (TEM) was prepared by drop-coating the Ag nanoparticles solution onto carbon-coated copper grid. The film on the grid was allowed to dry prior to the TEM measurement in a JEOL TEM-2010 instrument (JEOL Ltd., Akishima-shi, Japan). Solution pH values were measured by Mettler Toledo's pH meter (Mettler-Toledo Inc., Columbus, OH, USA).

### Preparation of plant extracts

Fresh and healthy leaves of Tulsi (*O. sanctum*), *C. limon*, and *J. adhatoda* were collected, washed thoroughly with double-distilled water, and were then cut into small pieces. A 10 g of finely cut pieces of leaves of each plant were then transferred into three different 250-mL beakers containing 100 mL distilled water each and boiled for 10 min. After cooling, the aqueous leaf extract obtained from the three different plants were filtered through ordinary filter paper, the filtrates were collected in three separate 100-mL volumetric flasks, and these 10% broth solutions were stored in a refrigerator for further use. On the dilution of the respective mother extract (10%) with requisite amount of distilled water, aqueous extracts of varying concentrations (7%, 5%, and 3%) were used in the present work.

### Synthesis of AgNPs and evaluation of reducing potential of the extracts

**Figure 1:**
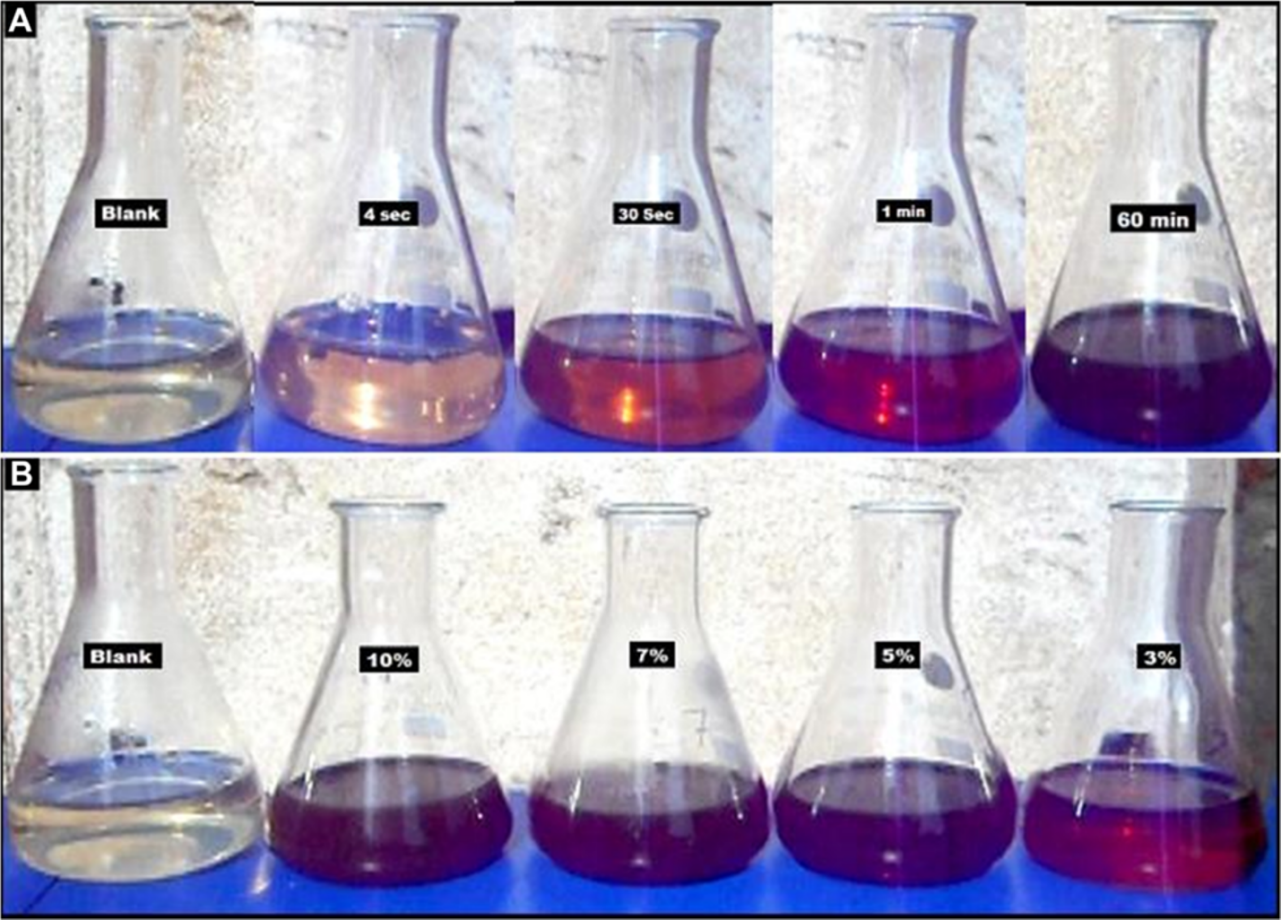
**Optical image. (A)** Gradual color change for the formation of AgNPs by 7% of *O. sanctum* leaf extract at different time intervals. **(B)** AgNPs formation with *O. sanctum* leaf extract at different concentrations (10%, 7%, 5%, and 3%) measured at 60 min.

*O. sanctum* leaf extracts of varying concentrations (10%, 7%, 5%, and 3%) were then transferred (5 mL each) into four different 100-mL conical flasks containing 45 mL of 10^−3^ M silver nitrate solution so as to make their final volumes to 50 mL each. The resulting solutions were kept under direct sunlight; gradual color change was then noted as an indication of silver nanoparticle formation (Figure 1) confirmed by UV-vis spectrophotometric studies at a regular time interval. Similar procedure was followed also for the other plant extracts.

### Analysis of bioreduced silver nanoparticles

#### UV-vis spectroscopy

To observe the optical property of biosynthesized silver nanoparticles, samples were periodically analyzed with UV-vis spectroscopic studies (Thermo Scientific Spectrascan UV 2700) at room temperature operated at a resolution of 1 nm between 200 and 600 nm ranges.

#### Transmission electron microscopy

TEM was performed for characterizing the size and shape of biosynthesized silver nanoparticles using JEOL TEM-2010 operated at an accelerating voltage of 300 kV. Prior to analysis, AgNPs were sonicated for 5 min, and a drop of appropriately diluted sample was placed onto carbon-coated copper grid. The liquid fraction was allowed to evaporate at room temperature.

### Microorganisms

The bacterial strains used in this study were procured from Microbial Type Culture Collection (MTCC), Institute of microbial technology, Chandigarh, India. The bacterial strains used belonged to both Gram-positive and Gram-negative categories. *Bacillus subtilis* MTCC121 was an endospore former; *Staphylococcus aureus* MTCC96, *Staphylococcus epidermidis* MTCC2639, and *Listeria monocytogenes* MTCC657 were Gram-positive bacteria; *Salmonella typhimurium* MTCC98, *Escherichia coli* MTCC1667, and *Pseudomonas aeruginosa* MTCC741 were human pathogenic Gram-negative bacteria; and *Pantoea ananatis* MTCC2307 was a plant pathogenic Gram-negative bacteria.

### Antimicrobial spectrum

Antimicrobial spectra for each concentration (5%, 7%, and 10%) of aqueous leaf extracts of *O. sanctum* as well as silver nano formed by these extracts upon sunlight induction were studied against four Gram-positive and four Gram-negative bacteria described above. Initially, 50 μL of each of the above samples were tested by agar well diffusion for antimicrobial screening [35]. Later, it was confirmed by colony-forming unit CFU counting method after serial dilution of the bacterial cultures under different treatments.

### Antimicrobial mode of action

Antimicrobial mode of action of the silver nano formed by 7% leaf extracts was studied against one Gram-positive *S. aureus* and one Gram-negative *P. aeruginosa*. The study was performed by applying 7% silver nano to the actively growing cultures of the bacteria followed by counting their CFU at regular intervals.

## Results and discussion

### UV-vis absorbance studies

**Figure 2:**
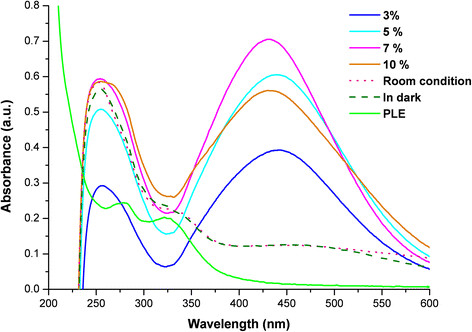
**UV-visible spectra for different concentrations of** ***O. sanctum*** **Linn. leaf extract (PLE) with 10** ^**−3**^ **M AgNO**_**3**_ **measured at 60 min.**

**Figure 3:**
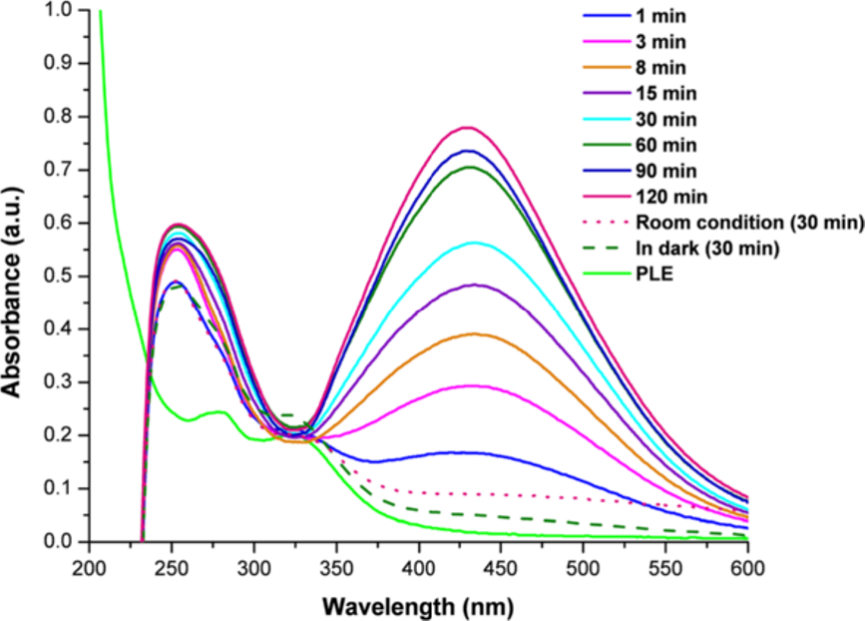
**UV-visible spectra for 7% aqueous** ***O. sanctum*** **Linn. leaf extract (PLE) with 10** ^**−3**^ **M AgNO**_**3**_ **at different time-intervals.**

The addition of fresh leaf extract of *O. sanctum* to silver nitrate solution resulted gradual color change of the solution from transparent to pale yellow, yellow, reddish, and finally to wine-red color due to the production of silver nanoparticles (Figure 1). These color changes arise because of the excitation of surface plasmon vibrations with the silver nanoparticles [44]. Initially, bioproduction of AgNPs was studied with different concentrations (3%, 5%, 7%, and 10%) of aqueous leaf extract of *O. sanctum* with 10^−3^ M AgNO_3_ solution. UV-visible spectra (Figure 2) indicated the formation AgNPs using these different concentrations of plant leaf extracts on exposure to direct sunlight for a time span of 60 min, and 7% plant leaf extract yielded the best result exhibiting the highest absorption band at 430 nm as a result of surface plasmon resonance (SPR) of silver nanoparticles. Now, we studied the UV-vis absorbance of the reaction mixture with 7% of *O. sanctum* leaf extract with varying time intervals of 1, 3, 8, 15, 30, 60, 90, and 120 min (Figure 3). It was observed that bioreduction of silver ions into nanoparticles started within 3 min and reached at an optimum level within 30 min (optimum absorption at 430 nm), thereby indicating rapid biosynthesis of silver nanoparticles; the absorption band measured at 60 min did not achieve much hike when measured at 90 and 120 min intervals. Broadening of the absorption peak at 430 nm with increase in time indicated the polydispersity of the nanoparticles.

**Figure 4:**
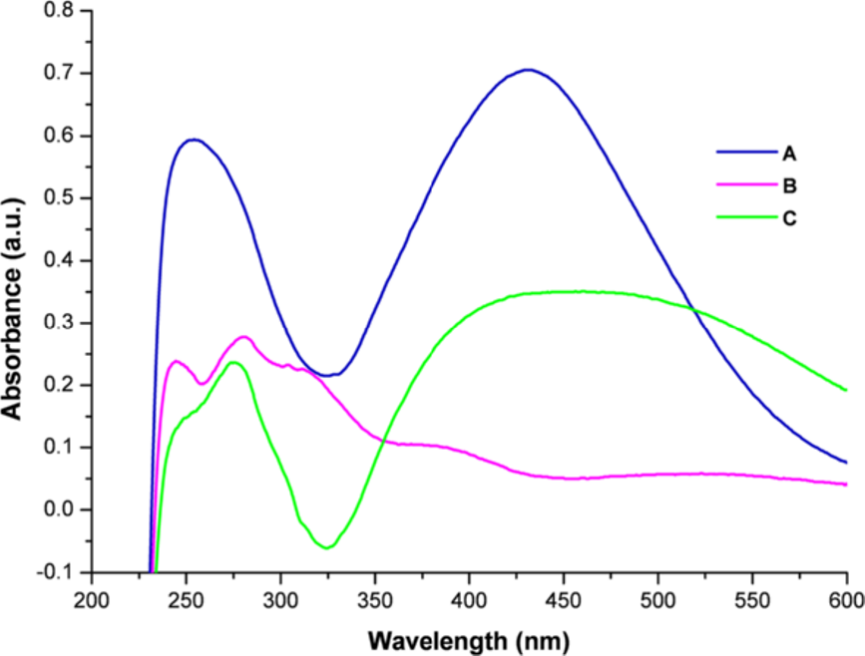
**UV-visible spectra of 7% aqueous leaf extract (PLE) of three different plants.** (Curve A) *Ocimum sanctum* Linn. (Curve B) *Citrus limon* L. (Curve C) *Justicia adhatoda* L. with 10^−3^ M AgNO_3_ measured at 60 min.

We also verified two other plant leaf extracts such as *C. limon* and *J. adhatoda* (7% aqueous extracts) to compare the results obtained from *O. sanctum* of the same concentration. The experimental results indicated a clear difference in their efficacy in producing silver NPs; aqueous (7%) leaf extract of only *O. sanctum* produced silver NPs reacting with 10^−3^ M AgNO_3_ solution giving rise to the plasmon band at 430 nm (Figure 4).

### TEM analysis

**Figure 5:**
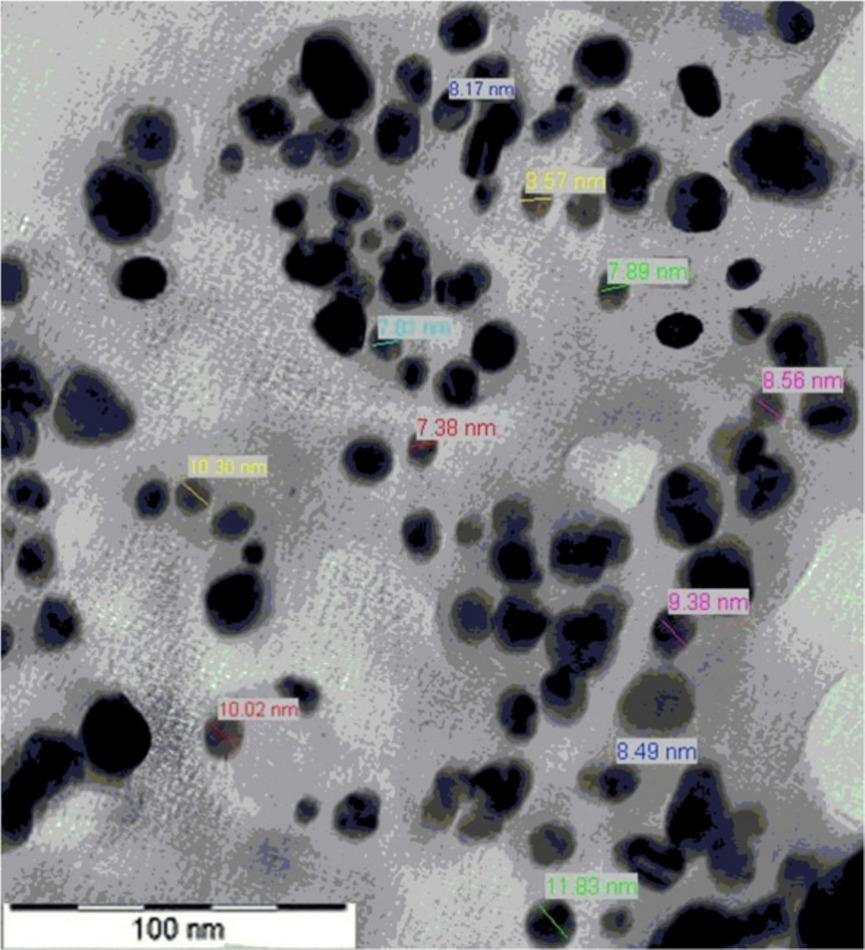
**TEM image of biosynthesized silver nanoparticles using** ***O. sanctum*** **leaf extract at 100 nm scale.**

While the UV-vis absorption spectral studies provided strong evidence for the formation of silver nanoparticles and their growth kinetics, the shape and size of the resultant nanoparticles are elaborated with the help of TEM analysis. The TEM image at 100-nm scale of the prepared silver nanoparticles formed by 7% aqueous leaf extract of *O. sanctum* under the influence of direct sunlight is presented in Figure 5. It was observed that Ag nanoparticles were circular in shape with maximum particles in size range within 7 to 11 nm. This particle size range also received support from the appearance of UV-vis absorption band at 430 nm [45].

### Bioreduction and stabilization of silver nanoparticles by *O. sanctum* fresh aqueous leaf extract: chemical perspective

**Scheme 1:**
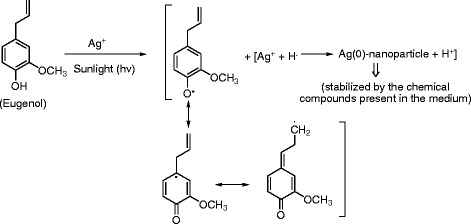
**Plausible photo-induced bioreduction of silver ions to silver nanoparticles by** ***O. sanctum*** **fresh leaf extract.**

From the GC-MS results as reported in literature [46],[47], *O. sanctum* fresh aqueous leaf extract is found to contain a variety of organic compounds among which the major components are eugenol, β-caryophyllene, β-elemene, cyclopropylidene, carvacrol, linalool, germacrene, etc*.* These chemical constituents are supposed to be responsible for photo-induced bioreduction of silver metal ions followed by stabilization of the nanoparticles formed. Since eugenol is the predominant member among the chemical constituents, we do assume a plausible mechanism for the rapid photo-induced bioreduction process (Scheme 1). On sunlight irradiation, the phenolic O-H bond undergoes homolytic cleavage to form hydrogen radical that eventually transfers its electron to a silver ion (Ag^+^)-generating silver nanoparticle. The oxygen radical part attains stabilization in the solution through extended conjugation. Hence, H^+^ ions are to be formed in the medium and their increasing concentration would affect the pH of the resulting Ag(NPs)-PLE medium. To verify our postulate, we then measured the pH of all types of solutions at 28°C: 1 mM AgNO_3_ solution (pH 6.54), PLE (pH 5.7), Ag(NPs)-PLE (sunlight, pH 4.8), Ag(NPs)-PLE (room temperature, pH 5.03). To our delight, the simple pH data are quite consistent with our postulation. Due to the presence of phenolic compounds like eugenol, the plant fresh leaf extract recorded moderately acidic pH at 5.7 that became lowered to pH 4.8 on adding silver nitrate solution (1 mM) under sunlight. However, at room temperature, Ag-NP formation is relatively much slower and not so prominent (pH 5.03).

### Antimicrobial analyses

Antimicrobial analyses were performed using three different (5%, 7%, and 10%) concentrations of aqueous leaf extracts of *O. sanctum* as well as the silver nanoparticles formed by them upon sunlight induction from a concentration of 10^−3^ M AgNO_3_ solution. Only AgNO_3_ solution at the same concentration was also tested against the bacteria for comparison, and an untreated control of bacterial cultures were also performed. The agar well diffusion experiment was run to perform a qualitative antimicrobial screening of the samples where clear inhibition zones of varying sizes were observed against all the eight bacteria tested. The other plant extracts at these concentrations did not produce any inhibition zones against these bacteria and are thus not considered for further antimicrobial studies. The preliminary qualitative observations obtained by agar well diffusion were verified quantitatively by CFU count method after treating each bacterial culture with the plant extracts as well as the silver nano formed by them after proper dilution and plating them on their suitable growth media and incubating either at 35°C or 28°C (*P. ananatis*) for overnight. It has been found that silver nano formed due to sunlight induction produced best result at 7% concentration against the bacteria tested. Its effect was more pronounced on non-endospore-forming Gram-positive *S. aureus* compared to other bacteria and stopped complete growth of the bacterium. The nanoparticles formed also showed prominent inhibitory activity on other bacteria at this concentration and also the other concentrations but with slightly lower degrees (Table 1). The results in Table 1 clearly depict its strong inhibitory action on the bacteria. This nanoparticle produced remarkable activity on Gram-positive bacterial strains and in particular the non-endospore forming *S. aureus*, *S. epidermidis*, and *L. monocytogenes.* The Gram-negative bacteria were also killed by the silver nano effectively but not at the same pace with Gram-positive members. Only AgNO_3_ killed the bacteria efficiently from a range of 6 × 10^8^ to 1.5 × 10^9^ CFU to 0.5 × 10^2^ to 1.5 × 10^3^ CFU. Leaf extracts of *O. sanctum* also could kill the bacteria but with lesser efficiency than the 10^−3^ M silver nitrate solution. The silver nano formed by the leaf extract at its different concentrations showed maximum efficiency, and in particular, the 7% aqueous leaf extract imparted the best antimicrobial action indicating the definite role of tulsi extract as well in enhancing the antibacterial potential. Interestingly, the observed antibacterial potential of AgNPs, formed by 7% aqueous tulsi leaf extract upon sunlight exposure, is even higher than almost the same size of AgNPs produced by the conventional methods [48],[49]. This is worthy to mention herein that 7% aqueous tulsi leaf extract afforded AgNPs of relatively smaller than those formed by the extracts of other concentrations (3%, 5%, and 10%) tested.

**Table 1:** **Count of colony forming units of different pathogenic bacteria and the AgNPs**

Microorganism	Control (untreated)	10^−3^M AgNO_3_Solution	5% PLE	AgNPs formed by 5% PLE	7% PLE	AgNPs formed by 7% PLE	10% PLE	AgNPs formed by 10% PLE
*Staphylococcus aureus*	1.2 × 10^9^	0.5 × 10^2^	1.4 × 10^2^	0.01 × 10^2^	1.7 × 10^2^	0	0.7 × 10^2^	0.02 × 10^2^
*Bacillus subtilis*	6.0 × 10^8^	1.0 × 10^3^	5.2 × 10^3^	0.5 × 10^2^	4.8 × 10^3^	0.3 × 10^2^	3.5 × 10^3^	0.3 × 10^2^
*Listeria monocytogenes*	7.0 × 10^8^	2.0 × 10^2^	1.2 × 10^3^	0.1 × 10^2^	9.0 × 10^2^	0.09 × 10^2^	5.6 × 10^2^	1.0 × 10^2^
*Staphylococcus epidermidis*	1.5 × 10^9^	2.0 × 10^2^	5.0 × 10^2^	0.8 × 10^2^	3.7 × 10^2^	0.05 × 10^2^	3.0 × 10^2^	0.08 × 10^2^
*Salmonella typhimurium*	8.0 × 10^8^	1.5 × 10^3^	6.0 × 10^3^	0.8 × 10^2^	5.5 × 10^3^	0.5 × 10^2^	4.5 × 10^3^	0.7 × 10^2^
*Pseudomonas aeruginosa*	8.5 × 10^8^	6.0 × 10^2^	7.2 × 10^3^	0.4 × 10^2^	7.0 × 10^3^	0.04 × 10^2^	6.9 × 10^3^	0.4 × 10^2^
*Escherichia coli*	8.0 × 10^8^	1.3 × 10^3^	2.0 × 10^3^	1.2 × 10^2^	1.5 × 10^3^	0.8 × 10^2^	8 × 10^2^	0.9 × 10^2^
*Pantoea ananatis*	8.5 × 10^8^	7.0 × 10^2^	4.0 × 10^3^	0.06 × 10^2^	1.7 × 10^3^	0.04 × 10^2^	9 × 10^2^	0.05 × 10^2^

After overnight growth upon treatment with different concentration of aqueous leaf extract of *Ocimum sanctum* and the AgNPs (dispersed in the aqueous leaf extract) formed by them upon sunlight induction. AgNPs, silver nanoparticles dispersed in the aqueous leaf extract; PLE, plant leaf extract.

**Figure 6:**
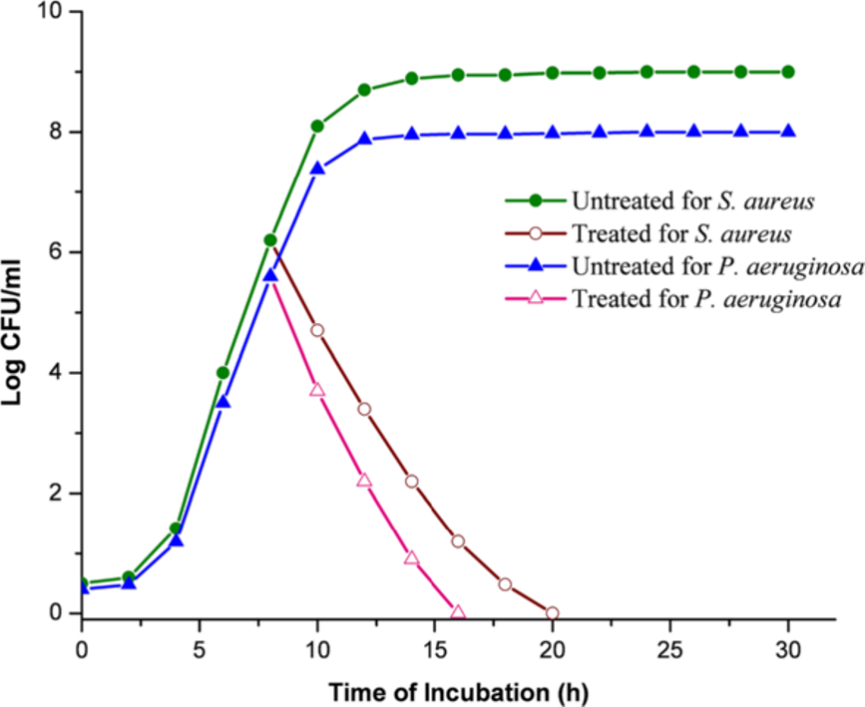
**Effect of the treatment of actively growing cells.** With AgNPs (dispersed in the aqueous leaf extract) formed by 7% leaf extracts of *O. sanctum* on growth pattern of *Staphylococcus aureus* [(●—●) for untreated and (○—○) for treated] and *Pseudomonas aeruginosa* [(▲—▲) for untreated and (Δ—Δ) for treated]. All values are means of three sets of experimental data.

Figure 6 indicates a clear bactericidal mode of action when silver nanoparticles (dispersed in 7% aqueous leaf extract) formed by 7% fresh leaf extract of *O. sanctum* were applied to the actively growing cultures of *S. aureus* and *P. aeruginosa.* A sharp decline in CFU counts from 2 × 10^6^ and 6 × 10^5^ to zero counts with time was observed for *S. aureus* and *P. aeruginosa*, respectively, upon treatment of the silver nanoparticles (dispersed in 7% aqueous leaf extract). The experimental outcomes unequivocally suggest a potent growth inhibitory activity of the nanoparticles upon both the microorganisms; however, the test material exhibited stronger activity against *P. aeruginosa* than that against *S. aureus*.

## Conclusion

In conclusion, we have developed a very simple, efficient, and robust practical method for the synthesis of silver nanoparticles using aqueous leaf extract of *O. sanctum* (Tulsi) as both reducing and capping agent, under the influence of direct sunlight. The biosynthesis of silver nanoparticles making use of such a traditionally important medicinal plant without applying any other chemical additives, thus offers a cost-effective and environmentally benign route for their large-scale commercial production. The nanoparticles formed are very effective in killing a number of bacteria in a bactericidal mode that include endospore formers, food spoilage pathogens, human as well as plant pathogenic members thus indicating their importance in controlling the growth of such microorganisms.

## Authors’ original submitted files for images

Below are the links to the authors’ original submitted files for images. Authors’ original file for figure 1 Authors’ original file for figure 2 Authors’ original file for figure 3 Authors’ original file for figure 4 Authors’ original file for figure 5 Authors’ original file for figure 6 Authors’ original file for figure 7
