# Supplementary figures and images for: Sunlight-induced rapid and efficient biogenic synthesis of silver nanoparticles using aqueous leaf extract of Ocimum sanctum Linn. with enhanced antibacterial activity
Source: Org Med Chem Lett. 2014 Dec 29;4(1):18. doi: 10.1186/s13588-014-0018-6 (PMC4297304; doi:10.1186/s13588-014-0018-6)

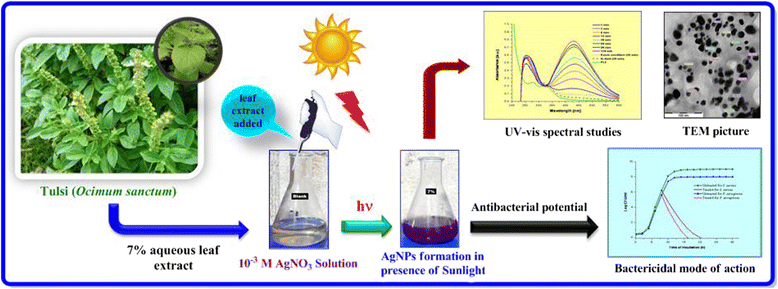

Supplement: Supplementary file 1 — Authors’ original file for figure 1 [file 13588_2014_18_MOESM1_ESM.gif]

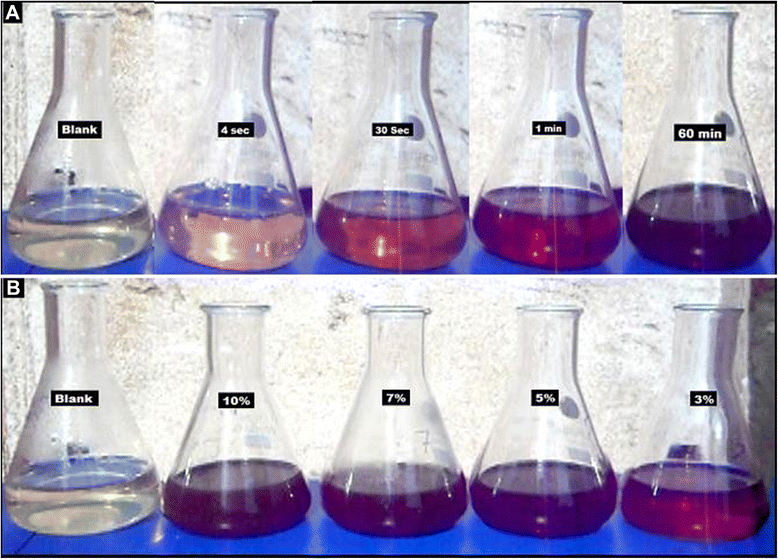

Supplement: Supplementary file 2 — Authors’ original file for figure 2 [file 13588_2014_18_MOESM2_ESM.gif]

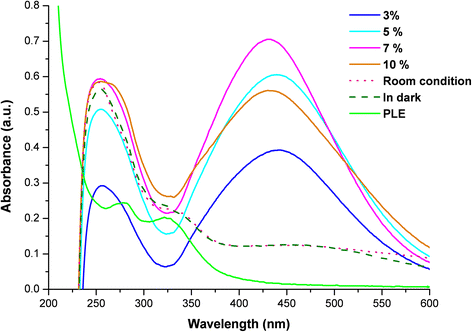

Supplement: Supplementary file 3 — Authors’ original file for figure 3 [file 13588_2014_18_MOESM3_ESM.gif]

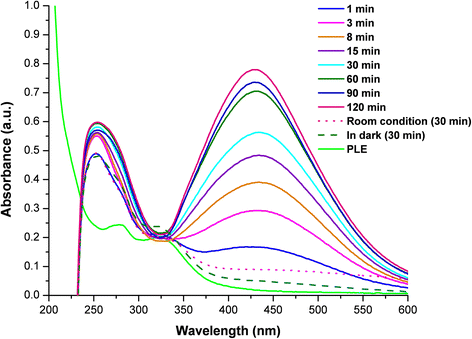

Supplement: Supplementary file 4 — Authors’ original file for figure 4 [file 13588_2014_18_MOESM4_ESM.gif]

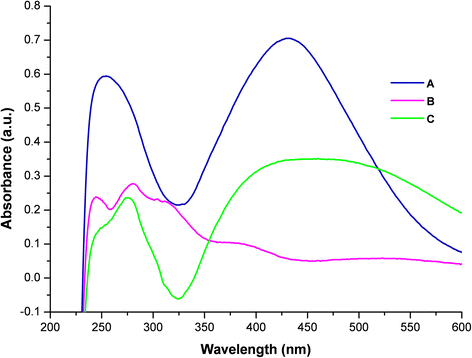

Supplement: Supplementary file 5 — Authors’ original file for figure 5 [file 13588_2014_18_MOESM5_ESM.gif]

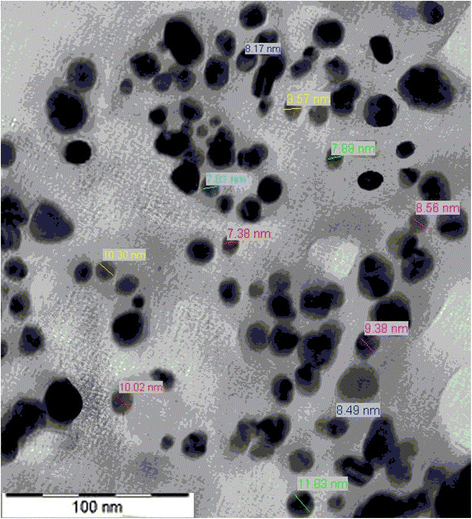

Supplement: Supplementary file 6 — Authors’ original file for figure 6 [file 13588_2014_18_MOESM6_ESM.gif]

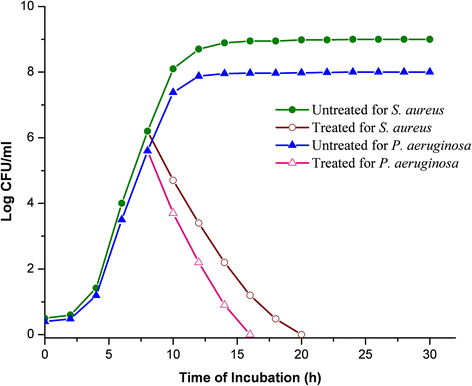

Supplement: Supplementary file 7 — Authors’ original file for figure 7 [file 13588_2014_18_MOESM7_ESM.gif]
